# Decoding diets: insights on ultra-processed food consumption among Lebanese adults from the updated LEBANese natiONal food consumption survey (LEBANON-FCS)

**DOI:** 10.3389/fnut.2024.1475223

**Published:** 2024-12-05

**Authors:** Maroun Khattar, Nikolaos Tzenios, Esraa Antar, Dana Malli, The Adults-Lebanon-FCS Group, Maha Hoteit

**Affiliations:** ^1^PHENOL Research Group (Public Health Nutrition Program-Lebanon), Faculty of Public Health, Lebanese University, Beirut, Lebanon; ^2^Faculty of Public Health, Charisma University, London, United Kingdom; ^3^Food Sciences Unit, National Council for Scientific Research of Lebanon (CNRS-L), Beirut, Lebanon

**Keywords:** adult, Lebanon, food consumption, NOVA classification, ultra-processed food

## Abstract

**Introduction:**

Ultra-processed foods are evident to play a role in the development of nutrition-related non-communicable diseases (NR-NCDs). There’s a scarcity of data in the Eastern Mediterranean Region (EMR) regarding ultra-processed food consumption, which highlights the need for such data in this region that is witnessing a nutrition transition. This study was conducted to assess the dietary pattern of Lebanese adults according to different degrees of food processing.

**Methods:**

A cross-sectional study involving a nationally representative sample (*n* = 444) of Lebanese adults (18–64 years) was conducted over the course of 5 months. A validated food frequency questionnaire and two non-consecutive 24-h recalls were used to collect the dietary intake. Sociodemographic and medical characteristics were collected using a validated questionnaire. Anthropometric measurements were taken by trained dietitians. Food items were classified according to the NOVA food classification system, and the percent contribution of every type of processing to the energy intake was calculated.

**Results:**

Ultra-processed foods contributed to the highest percent of energy intake in the sample (46.7%), followed by unprocessed and minimally processed foods (39.6%). Being male, and younger increased the odds of higher ultra-processed food intake; being employed and food secure increased the odds of a higher NOVA-UPF score. Compared with participants in Beirut, participants residing in North Lebanon and Akkar had higher odds of having a high UPF intake and lower odds of having a high NOVA-UPF score. The three most contributing food items to energy intake were ultra-processed (breads and breakfast cereals, fast foods, sweets and desserts). Compared to the unprocessed and minimally processed food diet fraction, the ultra-processed food diet fraction was significantly higher in sodium and thiamin and lower in proteins, fiber, and essential vitamins and minerals such as vitamins A, D, B12, folate, iron, calcium, and magnesium.

**Conclusion:**

UPF intake was the main contributor to TEI, and the intake was considerably higher among those who reported having renal disease, younger adults, and males. In comparison to the minimally processed diet fraction, the UPF diet fraction was found to have considerably greater levels of sodium and thiamin and lower levels of proteins, fiber, and essential vitamins and minerals. The study findings call for public health policies and interventions to encourage the consumption of minimally processed foods and decrease the consumption of ultra-processed foods, especially sweets and sweetened beverages, which are especially problematic.

## 1 Introduction

Non-communicable diseases (NCDs), such as cardiovascular diseases, cancer, diabetes, and chronic respiratory diseases, are on the rise globally and are nowadays the leading cause of global morbidity and mortality, exceeding communicable diseases (1). Based on the Centers for Disease Control and Prevention (CDC), NCDs lead to the death of 41 million people every year, which equates to 7 out of 10 deaths worldwide (2). NCDs impact people in all age categories, in all countries and regions. Although these illnesses are frequently linked to older age groups, data indicates that 17 million deaths from NCDs happen before the age of 70 (3). All age groups are susceptible to the risk factors that lead to NCDs, including poor eating habits, sedentary lifestyles, exposure to tobacco smoke, excessive alcohol use, and air pollution (3). For instance, excess salt/sodium intake leads to 1.8 million deaths per year, and insufficient physical activity leads to 830,000 deaths annually, as reported by the World Health Organization (WHO) (3). Additionally, the WHO considered unhealthy diets as one of the major risk factors leading to the development of chronic diseases (4). More profoundly, as reported by the Food and Agriculture Organization of the United Nations (FAO), there exists a solid link between the shift from consuming unprocessed/minimally processed foods to ultra-processed foods (UPFs) and the rise in overweight/obesity and other nutrition-related non-communicable diseases (NR-NCDs) (5). In fact, many studies are reporting that the consumption of UPFs is on the rise, with levels exceeding 45% of the daily caloric intake in the United States (U.S.), Canada, and the United Kingdom (UK) (6–8). UPFs can be defined as energy-dense, multi-ingredient, industrially formulated mixtures, with the majority being highly processed to be ready-to-eat with no preparation before consumption, such as breakfast cereals, fast foods, sweets, and packaged foods and beverages (5).

Several food classification systems are used to classify food items based on their degree of processing, among which the NOVA classification system received the most attention and is the only internationally accepted classification system that incorporates both formulation and processing using the “ultra” terminology (9). Within the NOVA classification system, food items are classified into four categories based on their degree of processing. Unprocessed foods, also referred to as “natural,” include the edible parts from animals (such as eggs, milk, offal, and muscle) or plants (such as fruits, roots, seeds, and stems). Minimally processed foods are those natural foods that have been modified by techniques like dehydrating, pulverizing, grinding… Since there’s not much difference between minimally processed and unprocessed meals, these two are considered as one group. Examples are fresh fruits and vegetables, dried fruits, grains, starchy roots and tubers, legumes, fresh or dried mushrooms, meat, poultry, fish and seafood, fresh juices with no added sugars, oats, eggs, pasteurized or powdered milk, plain yogurt, herbs and spices, water, tea and infusions, and coffee (10, 11). The second group is “processed culinary ingredients,” which are either obtained from group 1 or from nature by refining, milling, grinding, drying, or pressing. Examples include butter, ghee, oils obtained from nuts, seeds, or fruits (olives), lard, salt, added sugars, honey and molasses, uncooked raw pastas and starches and flours. Items of this group are usually used to prepare, season, or cook foods belonging to group 1. The third group is “processed foods” which are natural foods that are preserved through canning and bottling by adding salt, sugar, or oil, or by non-alcoholic fermentation when it comes to cheeses and breads. These include canned or bottled legumes, vegetables, fish, fruit in syrup (compote), roasted and salted nuts and seeds, and dried, smoked, or cured meat (ham, bacon), and fish (10, 11). The fourth group is called “ultra-processed foods” and refers to food compositions made primarily from materials intended for exclusive industrial use, usually by a sequence of industrial procedures and techniques. They usually have very few or no entire foods. Preservatives, stabilizers, emulsifiers, solvents, bulkers, binders, sweeteners, sensory enhancers, colors, and flavors, as well as processing aids and other additions, make up the bulk of the components in terms of numbers. They include many ready-to-eat products such as chocolate, candies, ice cream, carbonated beverages, sweetened fruit juices, sweetened milk, flavored yogurt, breakfast cereals, energy drinks and bars, cakes and pastries, biscuits, margarine, pre-packaged breads and buns, burgers, chicken nuggets and fish fingers, hotdogs, fries, pizza, pies, sauces, and instant coffee (10, 11).

In the Eastern Mediterranean Region (EMR), data on UPF consumption and its relation to chronic diseases is scarce, despite it being a region that is witnessing nutrition transition and increased prevalence of chronic diseases that account for over 80% of all deaths (12). In Lebanon, a country in the EMR, NCDs are the leading cause of death, representing 91% of all deaths (13). This is compounded by a nutrition transition to a dietary pattern high in saturated fats, added sugars, and sodium and low in essential vitamins and minerals, especially vitamins A, D, E, calcium, and iron, with adult women failing to meet their requirements and being at risk of anemia and osteoporosis (14). In addition, a study done in 2014 targeting adults in the Greater Beirut area revealed that 36.53% of the daily caloric intake of Lebanese adults comes from UPFs, the highest compared to minimally processed, processed culinary ingredients, and processed foods (15). Due to their role in developing NR-NCDs (5), it is crucial to assess the current consumption of UPFs in this population after the dietary shift that occurred in the recent years as a result of the economic collapse since 2019, the COVID-19 pandemic, and the Beirut blast in 2020. Such data is needed in Lebanon and the EMR given the scarcity of data on UPF consumption and its health-related outcomes in this region. For these reasons, the present study was done to (i) assess the contribution of minimally processed, processed, and ultra-processed foods to the daily consumption of Lebanese adults and (ii) investigate the correlates of UPFs consumption in the population.

## 2 Materials and methods

### 2.1 Study design

Over the period of 5 months (May to September 2022), a cross-sectional survey with a nationally representative sample (*n* = 444) of Lebanese adults (18–64 years) was carried out.

### 2.2 Sampling technique

In order to qualify as representative, the sample had to include at least 400 people. Population estimates from 2018 to 2019 were used to compute the sample size, which was then determined by applying the following formula: *n* = [p (1 − p)] × [(Z_∝/2_)^2^/(e)^2^].

**Figure 1:**
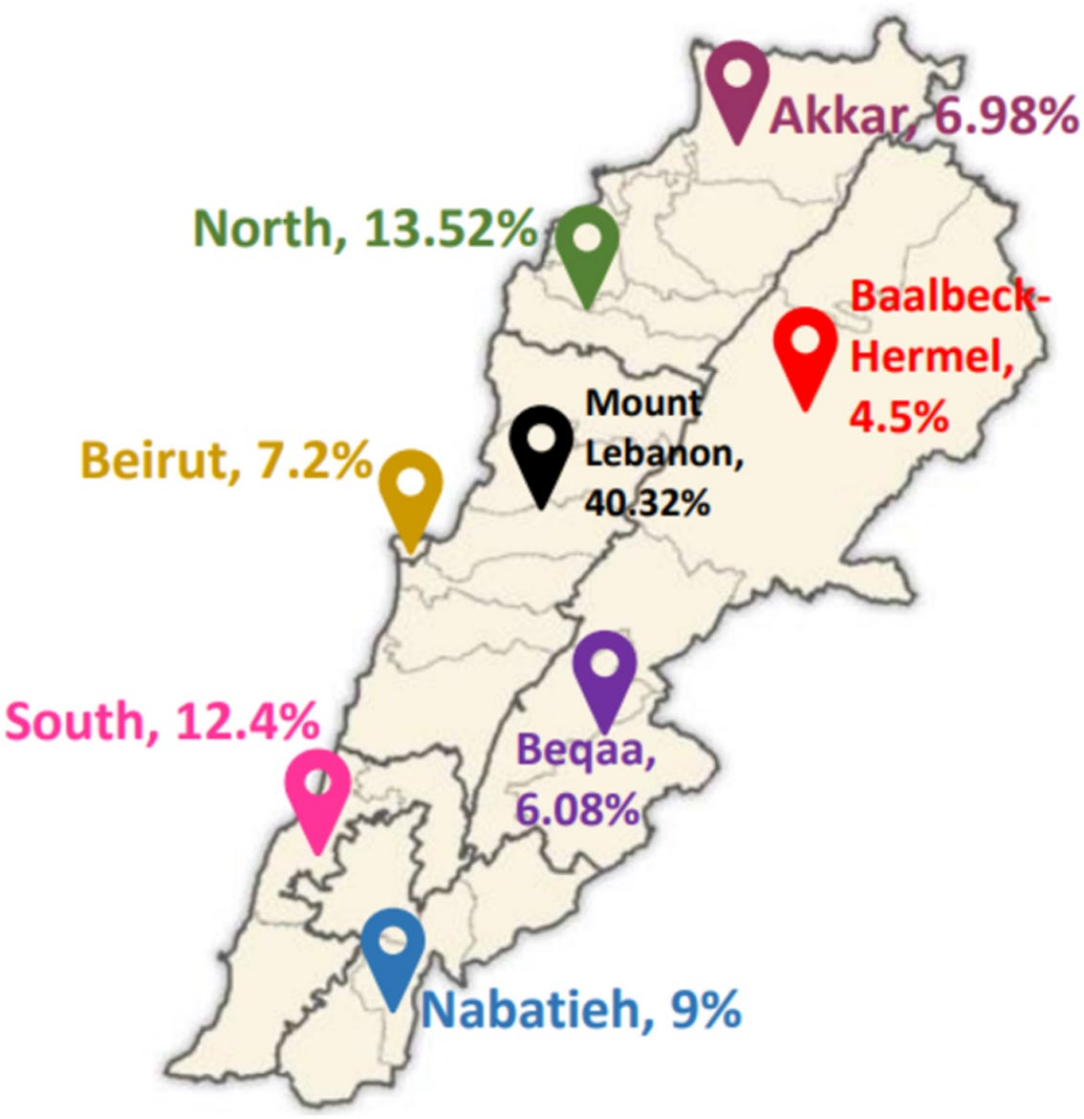
Distribution of study participants across the 8 Lebanese governorates. Adapted from George Saliba, Public domain, via Wikimedia Commons (17).

The above formula represents the sample size as ‘n’, the probability of adults not being able to take precautions against the diseases as 50% (represented by ‘p’), the standard error’s tolerated level as 5% (represented by ‘e’), and the reliability coefficient of the standard error at a 5% level of significance (=1.96) as ‘Z∝/2’ (16). The eight governorates of Lebanon served as the clusters, while the two genders served as the stratified groups in a stratified-cluster sampling combination. In order to provide a broad representation of Lebanese families, each household was limited to one participant. Participants were distributed over the 8 Lebanese governorates, as shown in Figure 1 (17).

### 2.3 Data collection

#### 2.3.1 Sociodemographic questionnaire

A pre-tested questionnaire was used to collect data by interviewing participants. It asked about the sociodemographic and medical characteristics of the study participants, such as their gender, age, weight, height, residency, education level, marital status, employment status, and the availability of chronic diseases. Plus, data regarding the household’s size and number of rooms within the households was recorded to compute the crowding index that reflects a household’s socioeconomic status (18).

#### 2.3.2 Food frequency questionnaire and 24-h recalls

To collect data about dietary consumption, trained dietitians carried out 30-min phone interviews with the study participants using a valid semi-quantitative food frequency questionnaire (FFQ) involving 157 food items (19) along with two non-consecutive 24-h recalls conducted during the same study period, and administered to all the participants. During the interviews, food items were recorded as either monthly, weekly, or daily for the FFQ and for the past day for the 24-h recalls. Monthly consumption was divided by 30 and weekly by 7 to unify all items as daily consumption (grams per day). After that, food items were classified in food groups (i.e., fruits, vegetables, dairy products, etc.) and the mean consumption was computed by adding the FFQ and 24-h recalls’ consumption, then dividing the value by 2 as follows: [value (FFQ) + value (24HR)]/2. In order to help participants remember what they ate and estimate the portions as accurately as possible, explanations and visual aid tools were provided. Food consumed was recorded as either monthly, weekly, or daily servings, then transformed into grams per day (g/d). For example, a monthly serving was divided by 30 to get the daily consumption.

#### 2.3.3 Anthropometric measurements

The weight and height of each participant were recorded three times to be as accurate as possible. Measurements were done based on standardized protocols and using calibrated equipment (20). After recording them thrice, the average was used to compute the Body Mass Index (BMI).

#### 2.3.4 Classification into unprocessed/minimally processed, processed culinary ingredients, processed and ultra-processed foods

After getting the amount of every food item consumed in g/d, items were classified as either “unprocessed/minimally processed,” “processed culinary ingredients,” “processed” or “ultra-processed” according to the NOVA classification. The NOVA classification framework gathers all foods according to their industrial processes’ nature, scope, and purposes. The processes involve biological, chemical and physical techniques used after separating foods from nature and before being consumed or prepared as meals and dishes (10, 11).

### 2.4 Ultra-processed food consumption score

Using the NOVA-UPF score adopted from Correa-Madrid et al. (21), a score of 1 was given if at least one food or drink from each category had been consumed, and a score of 0 if no product from the category had been consumed in order to calculate the individual score for UPF consumption. As such, individual scores can range from 0 to 23.

### 2.5 Nutrients and energy extraction

To extract the energy content and compare the nutrient profile of the ultra-processed diet fraction with the unprocessed and minimally processed diet fraction, items classified in each group were entered into “Nutritionist Pro” software (version 5.1.0, 2014, First Data Bank, Nutritionist Pro, Axxya Systems, San Bruno, CA, USA). This software allows the nutritional analysis of specific meals, menu items, and components used in recipes (22).

### 2.6 Data management and statistical Analysis

Microsoft Excel 2016 was used for data curation. The amount of food consumed was unified to daily consumption by dividing weekly consumption by 7 and monthly consumption by 30. After getting the amount of daily food consumption and classifying the food items according to NOVA, The Statistical Package for the Social Sciences (SPSS; Version 25.0, IBM Corp: Armonk, NY, USA) was used to compute the frequencies (N) and percentages (%) of the categorical variables and the mean and standard deviation (SD) of the continuous variables. Backward binary logistic regression analysis was performed to assess the correlates of UPF consumption. To do this, UPF consumption and score were divided into quartiles, and individuals in the highest quartile were considered to have a high intake/score, while those in the lowest quartile were considered to have a low intake/score. Comparison was done by comparing Q4 (high) to Q1/Q2/Q3 (labeled as “not high”) for high UPF intake/score correlates and by comparing Q1 (low) to Q2,Q3,Q4 (labeled as “not low”) for low intake/score correlates.

### 2.7 Ethical considerations

Patients who agreed to participate in the study signed a consent form before taking part. The study protocol was approved by the Ethics Committee of Al Zahraa University Medical Center (Approval Code: #57/2022; Approval Date: 5 October 2022) and was conducted in accordance with the Declaration of Helsinki (23).

## 3 Results

### 3.1 Characteristics of study participants

Tables 1 and 2 show the study participants’ sociodemographic and medical characteristics, respectively. Overall, the mean age of the participants was 34.13 (SD: 12.71) years, with more female than male participants (58.8% vs. 41.2%). Four out of 10 participants (40%) were residing in Mount Lebanon, and the majority of households were crowded (62.84%). About one-third (33.8%) of the participants had a normal BMI, while the majority were overweight and obese (61.9%). Three out of four participants (75%) reported not having a chronic disease, while one out of four (25%) reported having one or more chronic disease(s) with a significant difference between genders (*p-value* = 0.005). Of the participants who have chronic diseases, anemia (32.4%) and hypertension (30.6%) were the most prevalent, with a significant difference in disease types between genders (*p-value* = 0.004).

**Table 1:** Demographic and socioeconomic characteristics of the participants, overall and by gender.

		Overall (*n* = 444)	Male (*n* = 183)	Female (*n* = 261)	*p-value*
		*N* (%)	*N* (%)	*N* (%)	
Age Category	18 Years	20 (4.5%)	5 (25%)	15 (75%)	0.167
	19–30 Years	187 (42.1%)	78 (41.7%)	109 (58.3%)	
	31–50 Years	174 (39.2%)	79 (45.4%)	95 (54.6%)	
	51–64 Years	63 (14.2%)	21 (33.3%)	42 (66.7%)	
Residency (Governorate)	Akkar	31 (6.98%)	11 (35.5%)	20 (64.5%)	0.006^*^
	Mount Lebanon	179 (40.32%)	73 (40.8%)	106 (59.2%)	
	Beqaa	27 (6.08%)	5 (18.5%)	22 (81.5%)	
	North Lebanon	60 (13.52%)	33 (55%)	27 (45%)	
	Baalbek-Hermel	20 (4.5%)	10 (50%)	10 (50%)	
	South Lebanon	55 (12.4%)	30 (54.5%)	25 (45.5%)	
	Beirut	32 (7.2%)	10 (31.2%)	22 (68.8%)	
	Nabatiyeh	40 (9%)	11 (27.5%)	29 (72.5%)	
Marital Status	Single	202 (45.5%)	81 (40.1%)	121 (59.9%)	0.139
	Married	223 (50.23%)	97 (43.5%)	126 (56.5%)	
	Widowed	7 (1.57%)	0 (0.0%)	7 (100%)	
	Divorced	12 (2.7%)	5 (41.7%)	7 (58.3%)	
Crowding index	No crowding	165 (37.16%)	79 (47.9%)	86 (52.1%)	**0.028** ^*^
	Crowding	279 (62.84%)	104 (37.3%)	175 (62.7%)	
Number of children	0	217 (48.87%)	87 (40.1%)	130 (59.9%)	0.124
	1–3	159 (35.81%)	74 (46.5%)	85 (53.5%)	
	>3	68 (15.32%)	22 (32.4%)	46 (67.6%)	
Education level	Illiterate	3 (0.68%)	1 (33.3%)	2 (66.7%)	0.961
	School	175 (39.41%)	72 (41.1%)	103 (58.9%)	
	University	266 (59.91%)	110 (41.4%)	156 (58.6%)	
Employment Status	Unemployed	218 (49.1%)	40 (18.3%)	178 (81.7%)	**<0.001** ^*^
	Employed	226 (50.9%)	143 (63.3%)	83 (36.7%)	
Monthly salary changes after economic crisis	No Impact	129 (29%)	63 (48.8%)	66 (51.2%)	**<0.001** ^*^
	Decline in Salary	123 (27.7%)	51 (41.5%)	72 (58.5%)	
	Increase in Salary	70 (15.8%)	42 (60%)	28 (40%)	
	Already have no Salary	122 (27.5%)	27 (22.1%)	95 (77.9%)	
Household Monthly Income	None	39 (8.78%)	17 (43.6%)	22 (56.4%)	**<0.001** ^*^
	Less than 1.5 million LBP	58 (13.1%)	15 (25.9%)	43 (74.1%)	
	> = 1.5 million LBP	211 (47.5%)	79 (37.4%)	132 (62.6%)	
	<=300 USD	92 (20.72%)	41 (44.6%)	51 (55.4%)	
	More than 300 USD	44 (9.9%)	31 (70.5%)	13 (29.5%)	

^*^p-value < 0.05 is significant.

**Table 2:** Health characteristics of the study population, overall and by gender.

		Overall (*n* = 444)		Male (*n* = 183)		Female (*n* = 261)		
		Mean	SD	Mean	SD	Mean	SD	*p-value*
Weight (kg)		73.8	17.1	81.8	16.6	68.3	15.1	**<0.001** ^*^
Height (cm)		165.3	9.4	173.5	7	159.5	5.9	**<0.001** ^*^
Body mass index (kg/m^2^)		27	5.8	27.2	5.3	26.9	6.1	0.626
		*N*	%	*N*	%	*N*	%	
BMI classification	Underweight	19	4.3	4	21.1	15	78.9	0.084
	Normal	150	33.8	57	38	93	62	
	Overweight & Obese	275	61.9	122	44.4	153	55.6	
Disease status	No disease	333	75	150	45	183	55	**0.005** ^*^
	Having disease	111	25	33	29.7	78	70.3	
Disease type	Cardiovascular disease	14	12.6	5	15.2	9	11.5	**0.004** ^*^
	Diabetes	3	2.7	1	3	2	2.6	
	Hypertension	34	30.6	16	48.5	18	23.1	
	Kidney disease	4	3.6	2	6.1	2	2.6	
	Liver disease	1	0.9	1	3	0	0	
	Osteoporosis	14	12.6	1	3	13	16.7	
	Asthma/Respiratory diseases	12	10.8	2	6.1	10	12.8	
	Anemia	36	32.4	5	15.2	31	39.7	
	Others^*^	32	28.8	6	18.2	26	33.3	

^*^p-value < 0.05 is significant. BMI, Body Mass Index; cm, centimeters; kg, kilograms; m^2^, square meter; SD, Standard Deviation.

a Includes other self-reported diseases: (1) Allergies (seasonal, food, dust, skin); (2) Vertebral column problems; (3) Sarcoidosis; (4) Migraine; (5) Thyroid disease; (6) Gastrointestinal problems; (7) Psychological conditions; (8) Neurological conditions; (9) Hypovitaminosis D; (10) Hypocalcemia; (11) Iron deficiency; (12) Urinary tract infection; (13) Hypercholesterolemia; (14) Raynaud’s syndrome; (15) Varicose veins; (16) Autoimmune diseases; (17) Cancer; (18) Thrombosis; (19) Polycystic ovarian syndrome.

### 3.2 Consumption based on the NOVA classification

The mean dietary contribution of UPFs to total energy intake (%TEI) was 49.1% for male participants and 44.27% for female participants, with a significant difference between the two genders (*p-value* < 0.001). As for age groups, the highest %TEI from UPF consumption was for participants aged 18 years (53.07%), while older adults had the lowest %TEI from UPFs (43.39%), with a significant difference among the age categories (*p-value* = 0.041).

**Figure 2:**
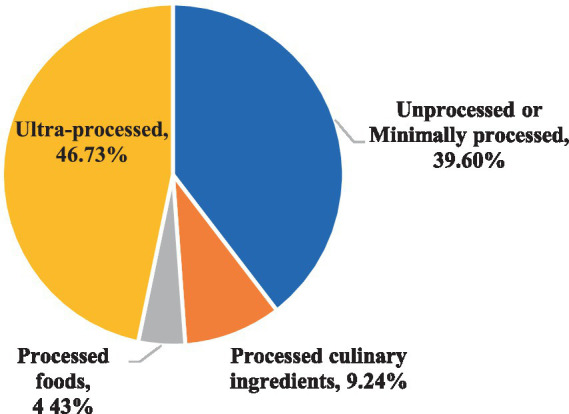
Distribution of total daily energy intake according to the NOVA classification.

Table 3 shows the dietary intakes of the different food groups in the study population based on the NOVA classification, which classifies food into four categories: unprocessed and minimally processed, processed culinary ingredients, processed foods, and UPFs. Overall, UPFs contributed to the highest percentage of total energy intake (%TEI) in the sample (46.73%), followed by unprocessed and minimally processed foods (39.6%; Figure 2). Based on food items, breads and ready-to-eat cereals contributed to the highest %TEI (19.92%), followed by fast food (10.4%) and desserts and sweets (9.59%), which means that the top three contributing items to energy intake are UPFs. In the unprocessed and minimally processed group, whole/refined grains and home-made dishes contributed to the highest %TEI (9.19%), followed by dairy products (7.38%) and fruits, fresh fruits juices, and dried fruits (7.05%). In the processed culinary ingredients group, olive oil and vegetable oils contributed to the highest %TEI (4.97%), followed by sugars, honey, and molasses (3.4%). As for the processed foods group, processed cheeses contributed to the highest %TEI (2.46%). Based on gender, the most contributing food items to %TEI for male participants were breads and cereals (20.96%), followed by fast food (11.33%) and desserts (9.74%), which are all UPF items. As for females, breads and cereals contributed to the highest %TEI (19.31%), followed by whole/refined grains and home-made dishes (9.87%) and fast food (9.15%). Male participants had significantly higher energy intake from meat and meat alternatives (*p-value* = 0.029), eggs (*p-value* = 0.025), unsalted nuts (*p-value* = 0.043), salted nuts and seeds (*p-value* = 0.029), canned and luncheon meats and sausages (*p-value* = 0.022), breads and ready-to-eat breakfast cereal (*p-value* < 0.001), fast food (*p-value* < 0.001), and sugar-sweetened beverages (*p-value* = 0.036) compared to female participants.

**Table 3:** Energy intakes of the different food groups in the study population based on the NOVA classification, overall and per gender.

		Total population (*n* = 444)		Male (*n* = 183)		Female (*n* = 261)		*p-value*
		Energy intake (Kcal/d)	Percent total energy intake	Energy intake (Kcal/d)	Percent total Energy Intake	Energy intake (Kcal/d)	Percent total Energy Intake	
Type of processing	Food groups/items	Mean	%TEI	Mean	%TEI	Mean	%TEI	
Unprocessed/Minimally processed	Dairy products	162	7.38%	150	6.33%	168	8.21%	0.174
	Fruits, fresh fruit juices, dried fruits	159.2	7.05%	161	6.79%	158	7.72%	0.848
	Fresh vegetables	80.99	3.69%	77.5	3.27%	83.2	4.06%	0.899
	Olives	12.84	0.58%	12.84	0.54%	12.84	0.63%	0.459
	Meat and meat alternatives (Red meat, poultry, fish)	125.13	5.7%	142	5.99%	113	5.52%	**0.029** ^*^
	Eggs	38.83	1.78%	46	1.94%	33	1.61%	**0.025** ^*^
	Legumes	75.49	3.45%	70	2.95%	80	3.91%	0.111
	Unsalted nuts	8.64	0.39%	13	0.55%	5	0.24%	**0.043** ^*^
	Whole/refined grains (pasta, rice, oats, quinoa), home-made dishes	201.64	9.19%	192	8.10%	202	9.87%	0.469
	Turkish coffee, tea and infusions	8.56	0.39%	10	0.42%	8	0.39%	0.129
	Water	0	0%	0	0%	0	0%	1
	**Total**	**868.86**	**39.6%**	**874.34**	**36.87%**	**863.04**	**42.16%**	0.752
Processed culinary ingredients	Olive Oil and vegetable oils	108.99	4.97%	120	5.06%	101	4.93%	0.067
	Butter, ghee, tahini	19.18	0.87%	21.16	0.89%	17.74	0.87%	0.246
	Sugar, honey and molasses	74.67	3.4%	80	3.37%	71	3.47%	0.236
	**Total**	**202.84**	**9.24%**	**221.16**	**9.33%**	**189.74**	**9.27%**	0.061
Processed foods	Salted nuts and seeds, peanut butter	19.42	0.88%	26.15	1.10%	14.48	0.71%	**0.029** ^*^
	Processed cheeses	53.92	2.46%	58.21	2.45%	52.12	2.55%	0.574
	Canned food (vegetables, fish) and cured or smoked meat (ham, bacon) and fish	23.97	1.09%	27.05	1.14%	21.48	1.05%	0.151
	**Total**	**97.31**	**4.43%**	**111.41**	**4.70%**	**88.08**	**4.30%**	0.086
Ultra-processed foods	Canned and luncheon meat, sausages	9.83	0.45%	12.9	0.54%	7.59	0.37%	**0.022** ^*^
	Breads^*^ and ready-to-eat cereals	437.1	19.92%	497.08	20.96%	395.36	19.31%	**<0.001** ^*^
	Condiments (mayonnaise, ketchup, sauces) and margarines	20.68	0.94%	22.36	0.94%	19.39	0.95%	0.211
	Fast food (sandwiches, pizza, fries, nuggets, pies)	228.35	10.4%	268.6	11.33%	187.3	9.15%	**<0.001** ^*^
	Desserts, sweets	210.43	9.59%	231	9.74%	187	9.13%	0.151
	Sugar-sweetened beverages	46.06	2.1%	56.7	2.39%	38.41	1.88%	**0.036** ^*^
	Chips, salty crackers	43.74	1.99%	44.1	1.86%	43.1	2.11%	0.894
	Spirits and alcohol	1.22	0.056%	0.97	0.04%	1.38	0.07%	0.128
	Instant coffee	28.16	1.28%	30.63	1.29%	26.53	1.3%	0.364
	**Total**	**1,025.57**	**46.73%**	**1,164.34**	**49.10%**	**906.06**	**44.27%**	**<0.001** ^*^

d, day; kcal, kilocalories; TEI, Total Energy Intake. ^*^“Breads” include different types (Arabic bread, white/brown bread, buns, baguette, toast…) and market brands of which many contain additives or other ultra-processed ingredients and were therefore considered as UPF.

### 3.3 UPF consumption based on sociodemographic characteristics

Table 4 shows the caloric intake of every UPF subgroup based on participants’ sociodemographic characteristics. Male participants significantly had higher caloric intake from ‘canned and luncheon meats and sausages’, ‘breads and ready-to-eat cereals’, fast food, and sugar-sweetened beverages compared to female participants. Younger adults had significantly higher consumption of fast food, desserts and sweets, sugar-sweetened beverages, and chips and salty crackers compared to older adults. As for crowding, non-crowded households had significantly higher caloric intake from ‘canned and luncheon meats and sausages’, fast food and condiments and margarines compared to crowded households. Our results also showed that employed participants significantly consumed more ‘canned and luncheon meats and sausages’, and fast food compared to unemployed participants, and food secure participants had significantly higher consumption of ‘canned and luncheon meats and sausages’, desserts and sweets, sugar-sweetened beverages and condiments and margarines compared to individuals with food insecurity. As for governorates, significant differences existed in the consumption of breads and ready-to-eat cereals, with individuals residing in the North having the highest consumption, and condiments and margarines, with individuals residing in Beirut having the highest consumption (see Supplementary Table S1 for *p*-values).

**Table 4:** Caloric intake of UPF subgroups according to sociodemographic characteristics.

Sociodemographic factors	UPF subgroups								
	Canned and luncheon meats and sausages	Breads and ready-to-eat cereals	Fast food	Desserts and sweets	Sugar-sweetened beverages	Chips and salty crackers	Spirits and alcohol	Instant coffee	Condiments & margarines
Gender									
Male	**12.9 ± 19.8**	**497.1 ± 271.5**	**268.6 ± 230.3**	231 ± 372.9	**56.7 ± 70.8**	44.1 ± 82.3	0.97 ± 40	30.6 ± 51.6	22.4 ± 21.7
Female	**7.6 ± 16.7**	**395.4 ± 251**	**187.3 ± 147.11**	187 ± 209.69	**38.4 ± 53.3**	43.1 ± 67.2	1.4 ± 17	26.5 ± 39.5	19.4 ± 25.2
Age									
18 Years	8 ± 11.2	452.65 ± 216.4	**334.4 ± 257.5**	**397.6 ± 734.1**	**105.8 ± 171**	**81.4 ± 112.8**	0.0 ± 0.0	39 ± 50.9	22.2 ± 18.4
19–30 Years	11.5 ± 21.6	448.4 ± 291.6	**222.1 ± 183.5**	**218.5 ± 264.1**	**41.4 ± 61.1**	**51.4 ± 85.3**	1.9 ± 15	31.8 ± 46.6	21.3 ± 20.1
31–50 Years	8.6 ± 16.4	446.8 ± 250.97	**217.6 ± 187.7**	**215.7 ± 250.9**	**47.45 ± 67.6**	**33.2 ± 45.7**	0.9 ± 10	26 ± 45.75	21.35 ± 32.1
51–64 Years	8.75 ± 11.6	372 ± 220.3	**187.9 ± 181.7**	**168.2 ± 181.3**	**37.1 ± 59.4**	**35.9 ± 79**	0.38 ± 83	20.2 ± 33.3	16.7 ± 12.1
Marital Status									
Married	9.8 ± 19	439.4 ± 245.85	235.2 ± 198.7	220.7 ± 303.8	46.4 ± 71.1	41 ± 63.4	0.3 ± 4.6	31 ± 49.4	21.8 ± 29.5
Not married	9.9 ± 17.2	434.8 ± 273.8	205.8 ± 180.1	216 ± 273.5	45.7 ± 74.4	46 ± 82.8	2.12 ± 16	25.3 ± 39.7	19.6 ± 118.6
Crowding Index									
Not crowded	**13.5 ± 21.6**	419.6 ± 241.6	**245.25 ± 206**	230.9 ± 243.	51.05 ± 81.5	50.8 ± 83.8	0.7 ± 5.8	30.3 ± 47.9	**24.9 ± 33.2**
Crowded	**7.7 ± 15.3**	447.5 ± 275.6	**205.9 ± 178.3**	210.9 ± 312.9	43.1 ± 66.95	39.1 ± 66.8	1.5 ± 14	26.9 ± 43	**18.2 ± 17.4**
Employment Status									
Unemployed	**7.3 ± 13.3**	414.5 ± 255	**200.8 ± 186.9**	216 ± 265.4	43.3 ± 78.9	43.1 ± 81.7	1.33 ± 13	24.8 ± 38.4	18.75 ± 27.5
Employed	**12.3 ± 21.5**	458.9 ± 271.5	**239.5 ± 191.4**	220.6 ± 310.3	48.7 ± 66.3	43.8 ± 65.3	1.1 ± 10	31.5 ± 50.2	22.6 ± 21.5
Food Security									
Food Secure	**12 ± 21.95**	425.7 ± 251.3	234 ± 198.3	**244 ± 270.9**	**52.9 ± 88.2**	48.2 ± 81.8	1 ± 10.2	30.1 ± 45.4	**23.4 ± 30.75**
Food Insecure	**7.4 ± 12.1**	449.9 ± 277.3	205.5 ± 179.6	**189.7 ± 305.6**	**38.5 ± 49.2**	38.2 ± 63.2	1.4 ± 13	26 ± 44.3	**17.8 ± 15**
Residency									
Beirut	7.7 ± 8.7	**362.2 ± 194.1**	190 ± 133.5	177.2 ± 210.5	47.1 ± 55.2	26.3 ± 34.6	0.0 ± 0.0	30.1 ± 43.1	**29.1 ± 57.9**
North & Akkar	8.4 ± 16.9	**504.6 ± 290.1**	213.5 ± 207.3	215.6 ± 308.1	49.5 ± 75.4	47.6 ± 86.9	1.7 ± 14.5	24.4 ± 38.3	**16.1 ± 19**
Mount Lebanon	10.2 ± 16.2	**429.5 ± 252.2**	226.5 ± 185.1	240.4 ± 347.5	46.1 ± 76	43.1 ± 70.7	0.2 ± 2.2	27.9 ± 42.9	**21.3 ± 17.7**
Beqaa	8.2 ± 14.7	**449.4 ± 296**	166.5 ± 133.3	208.2 ± 194.7	39 ± 45.8	65.9 ± 110.3	2.9 ± 20	41.1 ± 52.2	**15.2 ± 9**
South	12.1 ± 25.2	**405.9 ± 254.9**	253 ± 216	198.3 ± 196.1	45.8 ± 80.4	34.8 ± 45.7	2.2 ± 15.7	25.3 ± 50.4	**24 ± 26.8**

Values in Bold are significant.

### 3.4 Correlates of UPF consumption

#### 3.4.1 Socio-demographic determinants associated with high and low UPF intakes and NOVA-UPF scores

Results of the backward logistic regression models are shown in Table 5. Regarding high UPF intake, females had a lower likelihood of having high UPF intake compared with males (aOR = 0.48, 95% confidence interval (95% CI) = 0.306–0.76, and *p-value* = 0.002), people residing in North Lebanon and Akkar had higher odds of having high UPF intake compared with those residing in Beirut (aOR = 2.95, 95% CI = 1.07–8.05, and *p-value* = 0.035), and participants in the 31–50 age category had lower odds of having high UPF intake compared with those aged 18 years (aOR = 0.27, 95% CI = 0.09–0.75, and *p-value* = 0.012). As for low UPF intake, being a female increased the likelihood of having a low UPF intake by 72% (aOR = 1.72, 95% CI = 1.08–2.72, and *p-value* = 0.021).

**Table 5:** Sociodemographic determinants associated with high and low UPF intakes and NOVA-UPF scores.

Sociodemographic characteristics	UPF intake (as %TEI)								NOVA-UPF score							
	Determinants of high UPF intake				Determinants of low UPF intake				Determinants of High UPF score				Determinants of low UPF score			
	aOR	95% CI		*p*-value	aOR	95% CI		*p*-value	aOR	95% CI		*p*-value	aOR	95% CI		*p*-value
		Lower	Upper			Lower	Upper			Lower	Upper			Lower	Upper	
Sex																
Male (Reference)	-	-	-	-	-	-	-	-	-	-	-	-	-	-	-	-
Female	**0.48**	**0.30**	**0.76**	**0.002** ^*^	**1.72**	**1.08**	**2.72**	**0.021** ^*^	1.26	0.70	2.26	0.44	0.91	0.55	1.53	0.737
Age				0.064				0.082				0.583				0.095
18 Years (Reference)	-	-	-	-	-	-	-	-	-	-	-	-	-	-	-	-
19–30 Years	0.42	0.15	1.14	0.089	1.85	0.51	6.65	0.34	0.505	0.13	1.93	0.329	3.34	0.73	15.12	0.118
31–50 Years	**0.27**	**0.09**	**0.75**	**0.012** ^*^	1.95	0.54	7.04	0.31	1	0.407	2.47	0.993	4.15	0.91	18.87	0.066
51–64 Years	0.40	0.13	1.22	0.107	3.68	0.97	13.9	0.05	0.77	0.34	1.77	0.545	**5.86**	**1.23**	**28.01**	**0.027** ^*^
Marital Status																
Not Married (Reference)	-	-	-	-	-	-	-	-	-	-	-	-	-	-	-	-
Married	1.063	0.674	1.676	0.794	0.86	0.55	1.35	0.510	0.849	0.502	1.43	0.509	1.145	0.739	1.773	0.545
Crowding Index																
Not crowded (Reference)	-	-	-	-	-	-	-	-	-	-	-	-	-	-	-	-
Crowded	0.95	0.57	1.58	0.841	0.81	0.5	1.3	0.385	0.766	0.436	1.345	0.355	1.18	0.71	1.96	0.524
Employment Status																
Unemployed (Reference)	-	-	-	-	-	-	-	-	-	-	-	-	-	-	-	-
Employed	0.83	0.47	1.47	0.526	1.16	0.69	2.36	0.579	**1.76**	**1.04**	**2.98**	**0.03** ^*^	**0.63**	**0.41**	**0.97**	**0.034** ^*^
Education Level				0.279				0.531				0.86				0.616
Illiterate (Reference)	-	-	-	-	-	-	-	-	-	-	-	-	-	-	-	-
School	0.00	0.00	0.00	0.999	0.00	0.00	0.00	0.999	-	-	-	0.999	0.95	0.08	11.23	0.97
University	0.64	0.37	1.1	0.110	0.72	0.4	1.28	0.261	1.24	0.62	2.49	0.585	0.75	0.425	1.33	0.327
Food Security																
Food secure (Reference)	-	-	-	-	-	-	-	-	-	-	-	-	-	-	-	-
Food insecure	1.08	0.69	1.72	0.715	0.83	0.51	1.33	0.434	0.73	0.42	1.27	0.273	**1.63**	**1.03**	**2.54**	**0.034** ^*^
Residency				**0.001** ^*^				0.381				0.085				0.246
Beirut (Reference)	-	-	-	-	-	-	-	-	-	-	-	-	-	-	-	-
North & Akkar	**2.95**	**1.07**	**8.05**	**0.035** ^*^	2.75	0.87	8.69	0.085	**0.28**	**0.09**	**0.87**	**0.03** ^*^	2.144	0.89	6.97	0.081
Mount Lebanon	1.33	0.50	3.50	0.56	1.23	0.62	2.44	0.549	0.81	0.33	1.96	0.64	1.49	0.56	3.96	0.428
Beqaa	0.29	0.06	1.27	0.10	1.05	0.59	1.87	0.869	0.37	0.11	1.28	0.12	1.45	0.47	4.50	0.522
South	1.20	0.43	3.38	0.72	0.82	0.375	1.77	0.608	0.71	0.28	1.85	0.49	2.11	0.76	5.87	0.149

^*^p-value < 0.05 is significant. CI, Confidence Interval; aOR, Adjusted Odds Ratio; TEI, Total Energy Intake; UPF, Ultra-processed Foods. Variables entered in the models: Sex, age, marital status, crowding index, employment status, educational level, residency.

Concerning determinants of high NOVA-UPF score, employed participants had an increased likelihood of having a high NOVA-UPF score compared with unemployed participants (aOR = 1.76, 95% CI = 1.04–2.98, and *p-value* = 0.03). In addition, people residing in North Lebanon and Akkar were less likely to have a high NOVA-UPF score compared with those residing in Beirut (aOR = 0.28, 95% CI = 0.09–0.87, and *p-value* = 0.03). As for low NOVA-UPF score, being employed decreased the likelihood of having low NOVA-UPF score by 37% (aOR = 0.63, 95% CI = 0.41–0.97, and *p-value* = 0.034); being food insecure increased the likelihood of having low NOVA-UPF score by 63% (aOR = 1.63, 95% CI = 1.03–2.54, and *p-value* = 0.034); and participants in the 51–64 age category had higher odds of having low NOVA-UPF score compared with participants aged 18 years (aOR = 5.86, 95% CI = 1.23–28.01, and *p-value* = 0.027).

#### 3.4.2 Medical determinants associated with high and low UPF intakes and NOVA-UPF scores

Results of the regression models are shown in Table 6. Our results showed that participants who reported having kidney disease had higher odds of having a high UPF intake compared with participants who do not have kidney disease (aOR = 11.141, 95% CI = 1.086–114.28, and *p-value* = 0.042). Moreover, compared with participants having a normal body weight, being underweight decreased the likelihood of having a low NOVA-UPF score by 89% (aOR = 0.11, 95% CI = 0.015–0.86, and *p-value* = 0.036).

**Table 6:** Medical determinants associated with high and low UPF intakes and scores.

Medical characteristics	UPF intake (as %TEI)								NOVA-UPF score							
	Determinants of high UPF intake				Determinants of low UPF intake				Determinants of high UPF score				Determinants of low UPF score			
	aOR	95% CI		*p*-value	aOR	95% CI		*p*-value	aOR	95% CI		*p*-value	aOR	95% CI		*p*-value
		Lower	Upper			Lower	Upper			Lower	Upper			Lower	Upper	
BMI				0.728				0.178				0.28				**0.007** ^*^
Normal (Reference)	-	-	-	-	-	-	-	-		-	-	-		-	-	-
Overweight or Obese	0.71	0.22	2.26	0.561	1.06	0.33	3.45	0.914	0.43	0.09	1.98	0.28	0.2	0.026	1.59	0.13
Underweight	0.828	0.266	2.58	0.745	0.686	0.221	2.131	0.514	0.62	0.14	2.77	0.529	**0.11**	**0.015**	**0.86**	**0.036** ^*^
Cardiovascular disease																
No (Reference)	-	-	-	-	-	-	-	-	-	-	-	-	-	-	-	-
Yes	1.541	0.41	5.796	0.523	0.595	0.121	2.922	0.522	0.31	0.069	1.397	0.127	0.84	0.22	3.234	0.804
Diabetes																
No (Reference)	-	-	-	-	-	-	-	-	-	-	-	-	-	-	-	-
Yes	0	0	0	0.795	0.00	0.00	0.00	0.987	0.736	0.033	16.508	0.847	0.17	0.01	2.78	0.212
Blood pressure																
No (Reference)	-	-	-	-	-	-	-	-	-	-	-	-	-	-	-	-
Yes	0.99	0.43	2.281	0.981	1.681	0.764	2.695	0.196	0.853	0.305	2.39	0.763	1.75	0.715	4.277	0.221
Kidney disease																
No (Reference)	-	-	-	-	-	-	-	-	-	-	-	-	-	-	-	-
Yes	**11.141**	**1.086**	**114.280**	**0.042** ^*^	0.00	0.00	0.00	0.985	0	0	0	0.986	0.33	0.038	2.881	0.316
Osteoporosis																
No (Reference)	-	-	-	-	-	-	-	-	-	-	-	-	-	-	-	-
Yes	1.549	0.404	5.939	0.523	1.091	0.271	4.386	0.903	0.246	0.054	1.111	0.068	2.74	0.585	12.83	0.201
Asthma																
No (Reference)	-	-	-	-	-	-	-	-	-	-	-	-	-	-	-	-
Yes	0.662	0.145	3.013	0.594	3.114	0.983	9.863	0.053	0.379	0.082	1.1748	0.213	1.21	0.265	5.535	0.805
Anemia																
No (Reference)	-	-	-	-	-	-	-	-	-	-	-	-	-	-	-	-
Yes	0.411	0.151	1.122	0.083	0.813	0.327	2.018	0.655	2.096	0.638	6.884	0.223	1.79	0.663	4.882	0.249

^*^p-value < 0.05 is significant. CI, Confidence Interval; aOR, Adjusted Odds Ratio; TEI, Total Energy Intake; UPF, Ultra-processed Foods. Variables entered in the models: BMI, cardiovascular disease, diabetes, blood pressure, kidney disease, osteoporosis, asthma, anemia.

### 3.5 Comparison of the nutrient content between the UPF diet and the unprocessed and minimally processed foods diet

The nutrient profile’s comparison of the fraction of the diet from UPF and that from unprocessed and minimally processed foods is shown in Table 7. Significant differences were found between the two fractions for all the macro- and micro-nutrients assessed except for total fats. The UPF diet fraction was significantly lower in proteins and higher in carbohydrates. Additionally, the UPF diet fraction was significantly 1.78 times more concentrated in sodium and 1.3 times more concentrated in thiamin. Moreover, the UPF diet fraction was significantly 1.44 times less concentrated in calcium, 1.34 times less concentrated in iron, 2.84 times less concentrated in zinc, 2.63 times less concentrated in magnesium, 7.6 times less concentrated in vitamin A, 16 times less concentrated in vitamin D, 4.47 times less concentrated in vitamin C, 4.6 times less concentrated in vitamin K, 2.5 times less concentrated in riboflavin, 1.5 times less concentrated in niacin, 2 times less concentrated in vitamin B6, 2.5 times less concentrated in folate, 4.28 times less concentrated in B12, and 2.84 times less concentrated in fiber.

**Table 7:** Mean nutrient content in two diet fractions based on participants consumption.

	Fraction of the diet made up of ultra-processed foods	Fraction of the diet made up of unprocessed and minimally processed foods	*p-value*
Content of:			
Protein (% of total energy)	10.4	19.14	**<0.001** ^*^
Carbohydrates (% of total energy)	66.5	52.33	**<0.001** ^*^
Total fats (% of total energy)	23.1	28.53	0.287
Sodium (mg/1,000 kcal)	1,719.55	965	**<0.001** ^*^
Calcium (mg/1,000 kcal)	256.95	370.88	**<0.001** ^*^
Iron (mg/1,000 kcal)	6.03	8.06	**0.01** ^*^
Zinc (mg/1,000 kcal)	2.38	6.76	**<0.001** ^*^
Magnesium (mg/1,000 kcal)	90	236.75	**<0.001** ^*^
Vitamin A (RAE/1,000 kcal)	52.91	403.38	**<0.001** ^*^
Vitamin D (IU/1,000 kcal)	4.22	67.38	**<0.001** ^*^
Vitamin C (mg/1,000 kcal)	20.89	93.4	**<0.001** ^*^
Vitamin K (μg/1,000 kcal)	16.46	75.6	**<0.001** ^*^
Thiamin (mg/1,000 kcal)	0.64	0.49	**<0.001** ^*^
Riboflavin (mg/1,000 kcal)	0.43	1.08	**<0.001** ^*^
Niacin (mg/1,000 kcal)	7.38	11.32	**<0.001** ^*^
Vitamin B6 (mg/1,000 kcal)	0.57	1.14	**<0.001** ^*^
Folate (DFE/1,000 kcal)	113.99	285	**<0.001** ^*^
Vitamin B12 (μg/1,000 kcal)	0.61	2.61	**<0.001** ^*^
Fiber (g/1,000 kcal)	6.57	18.67	**<0.001** ^*^

^*^p-value < 0.05 is significant. DFE, Dietary folate equivalent; g, grams; IU, International Units; kcal, kilocalories; mg, milligrams; RAE, Retinol Activity Equivalent; μg, micrograms.

## 4 Discussion

To our knowledge, this is the first study in the EMR that used a nationally representative sample of adults to assess the consumption of minimally processed, processed, and ultra-processed foods based on a food classification system (NOVA). Our findings revealed that UPFs contributed to the highest %TEI in this population, representing 46.73%. Based on nationally representative individual-level dietary data that reported %TEI from UPFs in European countries, lower estimates were found in Belgium (24), where UPF consumption of adults aged 18–64 represented 29.6% of TEI. Lower estimates were also found in Portugal (24%) and France (31.1%) (25, 26), while higher estimates were found in the UK, where the %TEI of UPF consumption was 55% on average (8, 27). Nationally representative surveys in America revealed higher estimates in the U.S. and showed that the contribution of UPFs to %TEI represents 58% on average (6, 28, 29) while similar estimates were found in Canada (7), where adults aged 18–64 years had 45.7% of their TEI coming from UPF consumption. Concerning the UPF subgroups, breads and ready-to-eat cereals were the most consumed by our study population. Similarly to Colombia (30), while desserts were the most consumed UPF subgroup in Chile, Mexico and Brazil (31–33).

In our study, being a female decreased the odds of high UPF intake and increased the odds of having low UPF intake, indicating that female participants significantly consumed less UPF compared with male participants. Nationally representative surveys showed similar results in Brazil (34), Canada (7, 35), Korea (36), and Switzerland (37), where females had significantly lower consumption of UPFs than males. Different results were obtained in Spain (38) and Italy (39), where females had significantly higher consumption of UPFs. Concerning age, younger adults were found to consume more UPF compared to older adults, as older adults in our population had lower odds of having high UPF intake and higher odds of having a low NOVA-UPF score compared with those aged 18 years. Similar results were obtained in nationally representative surveys in Brazil (34), Australia (40), Barbados (41), France (26), and Mexico (32), where younger adults significantly consumed more UPF. The higher consumption of UPFs in younger adults might be explained by the fact that younger generations are more willing to try new and trendy foods compared to older generations, who prefer sticking to traditional and fresh foods (42).

In addition to gender and age, employment status, food security status, and residency were determinants of UPF in our population. Employed individuals had higher odds of having a high NOVA-UPF consumption score and lower odds of having a low NOVA-UPF score compared with unemployed individuals, indicating that employed individuals’ consumption of UPF is higher. Similar results were obtained in Brazil (43), where employed individuals had significantly higher consumption of UPF. As for food security, in our study, food insecure individuals had higher odds of having a low NOVA-UPF score, indicating a lower consumption of UPF. Different results were obtained in Canada and France, where higher levels of insecurity were associated with higher UPF intake (44). Concerning residency, participants residing in North Lebanon and Akkar had higher odds of having high UPF intake while lower odds of having a high UPF score compared with people residing in Beirut. This difference might be due to the fact that a person might consume more than one UPF item belonging to the same category and thus receive a lower UPF score despite having a high UPF intake as %TEI. In addition, North Lebanon and Akkar are the governorates with the highest prevalence of food insecurity (45); thus, participants from these governorates are less likely to have a variety of food choices, which might explain the decreased odds of a higher UPF score compared with those residing in Beirut.

In our population, participants who reported having kidney disease had significantly higher consumption of UPFs in our study. The current finding is alarming, as greater UPF consumption was shown to be associated with chronic kidney disease (CKD) progression, especially in early stages (stages 1–2) (46). In addition, there was an association between increased consumption of UPF and an increased risk of all-cause mortality in adults with CKD (46). Moreover, higher UPF intake was associated with a reduction in renal function in Spain (47). This highlights the importance of encouraging unprocessed and minimally processed foods over UPF consumption in patients with CKD as a prevention and treatment strategy and is a need in the Lebanese population, as revealed in our study.

When it comes to nutritional content, the UPF diet fraction in 1,000 kcal was shown to be significantly higher in sodium and lower in protein, fiber, and essential vitamins and minerals (such as magnesium, calcium, vitamins A, C, D, and B12). Similar findings were obtained in nationally representative surveys in the U.S. (for vitamins A, C, D, calcium, magnesium, and zinc), Canada (for vitamins A, C, D, B12, niacin, riboflavin, calcium, iron, magnesium, zinc, and sodium), Brazil (for vitamins D, B12, B6, niacin, iron, magnesium, and zinc), Taiwan (for vitamins A, C, D, B6, niacin, magnesium, calcium, and iron), and in the UK, Belgium, and Australia for sodium (10). Different findings were obtained in Brazil (10) for calcium, where the increased share of UPF increased calcium intake. For thiamin, a higher dietary content in the UPF fraction was shown in our study, while different results were obtained in Canada and Taiwan (10). For fiber content, similar findings were found in the U.S., UK, Canada, Mexico, Colombia, Brazil, Chile, and Australia (10), where increased UPF intake showed decreased dietary content of fiber and a probability of insufficient intake (<25 g/2,000 kcal). For proteins, similar findings were found in the U.S., UK, Canada, Mexico, Colombia, Brazil, Chile, and Taiwan, where a higher share of UPF was associated with lower protein intake (10). In short, a higher %TEI coming from UPF reflects lower intake of essential vitamins and minerals, eventually increasing the risk for micronutrient deficiencies and adverse health outcomes.

The findings of this study add the risk for kidney disease progression to the previous findings that assessed the dietary pattern of this population (14, 48) and further suggest that Lebanese adults, especially women, are at high risk of developing osteoporosis, anemia, and micronutrient deficiencies (hidden hunger). For instance, the UPF diet fraction was shown to be high in sodium and low in calcium, and when sodium is taken in high amounts, it interferes with calcium absorption and increases its excretion, especially when calcium intake is low (49). In addition, diets high in sodium were shown to be the leading cause of global deaths attributable to diet in a systematic analysis conducted in 2017 (50), highlighting the importance of limiting UPF intake. Consuming more fresh and minimally processed foods and decreasing the consumption of UPF (especially refined breads and breakfast cereals, fast food, sweets, and sugar-sweetened beverages in our population) might therefore improve the diet quality of Lebanese adults, especially for sodium, whose intake exceeded the RDA, and vitamins and minerals such as vitamins A, D, and B12, and calcium and iron, whose intakes were shown to be low in this population, with women being more deficient than men (14, 48).

Many countries assessed the association between UPF consumption and the dietary content of free or added sugar and saturated fats. A strong positive association of UPF consumption and free or added sugar content of the diets (or levels of added sugars exceeding 10% of the TEI) was shown in the eight countries that assessed this (U.S., Brazil, UK, Chile, Canada, Colombia, Australia, Mexico) (10). Additionally, a strong positive dose–response association was found between UPF consumption and saturated fat content in the diets (or levels exceeding 10% of the TEI) in all the countries that assessed it (U.S., Brazil, UK, Chile, Canada, Colombia, Australia, Mexico, Belgium, Taiwan) (10). Such findings are alarming, as UPF contributed to the highest %TEI in our sample, and since the dietary pattern of our population was shown to be high in refined carbohydrates, added sugars and saturated fats, with a high reliance on sweets (desserts, cakes, and pastries) and sugar sweetened beverages (carbonated beverages and sweetened fruit juices) (14), which are especially problematic as UPF (10).

UPF consumption is driven by several socio-cultural factors, including sex, age, employment status, education level, marital status, and food security status. In addition to the socio-cultural factors, exposure to UPF plays a significant role in driving consumption. Moreover, the type of food should be taken into consideration, as soft drinks and sugar-sweetened beverages are more concerning and pose more harm than other items (10). Given the significant and relatively quick rise in UPF sales and consumption, and their adverse health effects, lowering their use and altering the national food environment is of priority. As revealed in our study, Lebanese adults are at a high risk of developing NR-NCDs, especially anemia, kidney disease, and osteoporosis, as well as micronutrient deficiencies, and this is exacerbated by increased UPF consumption. In fact, an increased %TEI from UPF is associated with all-cause mortality, obesity, metabolic syndrome, depression, cardiometabolic diseases, frailty, irritable bowel syndrome, functional dyspnea, and cancer (51, 52), and this is threatening to the public health on the long-term. Targeted interventions are needed in the country to promote healthy dietary patterns and nutrition environments, and this can be done in many ways. For instance, public health policies and campaigns that highlight the risks posed by UPF and support food systems to make unprocessed and minimally processed foods as well as freshly prepared meals and dishes more available, affordable, and valued are needed. These foods include grains, starchy roots and tubers, legumes, and other plant-based foods. Moreover, it is crucial to restrict the marketing and exposure of UPF, especially to children and younger adults, and this can be done by creating policies that limit UPF in schools and public institutions. Additionally, it is important to consider front-of-package warning labels that identify ultra-processed products and regulate the food industry’s formulation and processing practices. As such, research priorities must focus on assessing the different drivers for UPF consumption, such as the difference in exposure between rural and urban settings, demographics, the type of food, as well as understanding the mechanisms of harm as UPF consumption is associated with many chronic diseases (51, 52). Importantly, evaluation studies need to concentrate on real purchases and consumption. In order to achieve the long-term objective of making healthier options the convenient ones, qualitative studies about what might alter eating norms in the population might serve as an efficient approach.

### 4.1 Strengths and limitations

The current study assessed the dietary pattern of Lebanese adults based on different degrees of food processing and is the first study in Lebanon and the EMR to do this on a nationally representative sample. Thus, this study contributes to the literature and addresses a crucial topic that has not yet been addressed nationally in the country and the region. In addition, the sample is representative, which allows the results of the study to be generalized to the whole population. Moreover, the use of a combination of FFQ and two non-consecutive 24-h recalls increases the accuracy of the results (53). However, the method used for data collection relies on memory and is therefore subject to recall bias as well as inaccuracies when estimating portion sizes. To decrease this probability, the trained dietitians provided visual tools to participants to help them recall and estimate portions as accurately as possible.

## 5 Conclusion

The consumption of UPFs contributes to the highest %TEI in Lebanese adults. Males, younger adults, and participants who reported having kidney disease had significantly higher UPF consumption. The UPF diet fraction was shown to be significantly higher in sodium and thiamin and lower in proteins, fiber, and essential vitamins and minerals compared to the minimally processed diet fraction. The current findings emphasize the need for public health policies and interventions that would encourage healthy eating behaviors in this population that rely heavily on UPF items, especially sweets, desserts, and refined grains that are low in fiber, essential vitamins and minerals, and high in sodium, saturated fat, and added sugars. The findings can also be used by stakeholders and policymakers to limit the exposure to UPF and encourage the consumption of unprocessed and minimally processed foods over UPF in the country and the region.

## Group members of the Adults-LEBANON-FCS group

Zahraa Fadlallah, Razan Khadra (Faculty of Public Health, Lebanese University); Mohamad Chahine, Omasyarifa Binti Jamal Poh (Kursk State University, Russia), Nikolaos Tzenios (Faculty of Public Health, Charisma University, London EC1V 7QE, United Kingdom), Elham Al Manasfi, Abdulrahman Chahine (Arab Group for Scientific Research, Beirut, Lebanon).

## Data Availability

The original contributions presented in the study are included in the article/Supplementary material, further inquiries can be directed to the corresponding authors.
