# Supplementary material for: Decoding diets: insights on ultra-processed food consumption among Lebanese adults from the updated LEBANese natiONal food consumption survey (LEBANON-FCS)
Source: Front Nutr. 2024 Dec 5;11:1475223. doi: 10.3389/fnut.2024.1475223 (PMC11656934; doi:10.3389/fnut.2024.1475223)
Supplement: Supplementary file 1 [file Table_1.DOCX]

| **Supplementary Table S1.** P-values of UPF subgroups consumption according to sociodemographic characteristics | | | | | | | | | |
| --- | --- | --- | --- | --- | --- | --- | --- | --- | --- |
| **Sociodemographic Characteristics** | **UPF subgroups** | | | | | | | | |
|  | Canned and luncheon meats and sausages | Breads and ready-to-eat cereals | Fast food | Desserts and sweets | Sugar-sweetened beverages | Chips and salty crackers | Spirits and alcohol | Instant coffee | Condiments & margarines |
| Gender | **0.019** | **<0.001** | **<0.001** | 0.114 | **0.009** | 0.892 | 0.717 | 0.341 | 0.200 |
| Age | 0.419 | 0.216 | **0.028** | **0.022** | **0.001** | **0.009** | 0.719 | 0.191 | 0.580 |
| Marital Status | 0.946 | 0.853 | 0.104 | 0.865 | 0.923 | 0.478 | 0.105 | 0.185 | 0.357 |
| Crowding Index | **0.001** | 0.284 | **0.035** | 0.483 | 0.267 | 0.107 | 0.455 | 0.438 | **0.006** |
| Employment Status | **0.004** | 0.077 | **0.032** | 0.867 | 0.439 | 0.929 | 0.832 | 0.115 | 0.100 |
| Food Security | **0.007** | 0.336 | 0.115 | **0.048** | **0.037** | 0.152 | 0.771 | 0.332 | **0.017** |
| Residency | 0.573 | **0.038** | 0.103 | 0.698 | 0.957 | 0.102 | 0.506 | 0.287 | **0.026** |
| Values in **Bold** are significant | | | | | | | | | |

Supplementary Material
